# An in-vivo study of BOLD laminar responses as a function of echo time and static magnetic field strength

**DOI:** 10.1038/s41598-021-81249-w

**Published:** 2021-01-21

**Authors:** Irati Markuerkiaga, José P. Marques, Lauren J. Bains, David G. Norris

**Affiliations:** 1 Donders Centre for Cognitive Neuroimaging, Radboud University, Nijmegen, The Netherlands; 2 Erwin L. Hahn Institute for Magnetic Resonance Imaging, 45141 Essen, Germany

**Keywords:** Attention, Perception, Sensory processing

## Abstract

Layer specific functional MRI requires high spatial resolution data. To compensate the associated poor signal to noise ratio it is common to integrate the signal from voxels at a given cortical depth. If the region is sufficiently large then physiological noise will be the dominant noise source. In this work, activation profiles in response to the same visual stimulus are compared at 1.5 T, 3 T and 7 T using a multi-echo, gradient echo (GE) FLASH sequence, with a 0.75 mm isotropic voxel size and the cortical integration approach. The results show that after integrating over a cortical volume of 40, 60 and 100 mm^3^ (at 7 T, 3 T, and 1.5 T, respectively), the signal is in the physiological noise dominated regime. The activation profiles obtained are similar for equivalent echo times. BOLD-like noise is found to be the dominant source of physiological noise. Consequently, the functional contrast to noise ratio is not strongly echo-time or field-strength dependent. We conclude that laminar GE-BOLD fMRI at lower field strengths is feasible but that larger patches of cortex will need to be examined, and that the acquisition efficiency is reduced.

## Introduction

The human iso-cortex is ~ 1.5–3 mm thick and is divided into six anatomical layers in most cortical regions. According to the canonical model of neural processing^1^, layers play a specific role in the processing within a cortical area and in the feed-forward/feedback connections between cortical regions. Therefore, there is a growing interest to acquire high-resolution functional MRI data to measure the layer specific response and understand the interactions between brain regions.

In MRI, high spatial resolution implies a reduced image Signal to Noise Ratio (SNR). Image SNR and temporal SNR (tSNR) increase with the strength of the main magnetic field^2^. Therefore, most layer specific BOLD-fMRI studies in humans in recent years have been performed at 7 T to obtain a higher sensitivity to activation^3–13^.

Many of these layer specific studies at 7 T further improved the signal to noise ratio (SNR) by integrating the signal over a patch of cortex at a given cortical depth. In an activated region, the larger the number of voxels that are integrated the better the sensitivity to activation will be, provided the noise is not coherent between voxels. That is the case if only thermal noise is present. Once physiological noise sources, i.e. those that result in signal modulations unrelated to neuronal changes induced by the functional task, become important, the signal variation will become correlated over voxels and sensitivity will not improve by summation of new voxels. In this physiological noise regime^2,14–17^ the sensitivity gain in moving to higher static field strengths will be lower than for the thermal noise regime.

This argumentation would in principle apply to all the forms of functional contrast so far explored for laminar fMRI at 7 T: gradient-echo (GE) BOLD^5,6,9–12,18–21^, spin-echo based BOLD^3,4,7,22^, VASO^23–25^ and SSFP^26^. Due to its robustness, high functional sensitivity, and speed of acquisition, GE-BOLD is a widely used approach in layer specific fMRI. An assessment of the potential value of GE-BOLD laminar fMRI at static field strengths below the gold standard of 7 T requires a characterisation of the laminar activation profile as a function of TE, and measurement of the relative sensitivity when data are acquired in the physiological noise regime.

We have previously measured activation profiles and noise characteristics at 7 T^8^. Here we repeat the 7 T study and extend this approach to 3 and 1.5 T. In order to perform a comparison based on BOLD signal properties alone, it is important to obtain largely distortion and dropout free images at all field strengths and echo times. This is achieved by measuring with a multi-echo FLASH sequence as the more common EPI image encoding scheme would suffer field-dependent distortion and signal loss. In this manuscript, first the laminar BOLD-dependent signal as a function of TE and relaxation time changes are compared across static magnetic field strengths. These features are independent of the temporal resolution. Subsequently, the noise in the signal, which is a feature that depends on the temporal resolution, is analysed to describe the noise regime for which the functional sensitivity results obtained here apply.

## Materials and methods

Nine healthy volunteers (4 female, 30 ± 4 years old) were scanned in three separate sessions at 1.5 T, 3 T and 7 T (Avanto, Skyra and Magnetom, respectively, Siemens Healthcare, Erlangen, Germany). The coil configurations used at 1.5 T and 3 T were the vendor-provided 32 Channel head coil for reception and the body coil for transmission. At 7 T, the single channel transmit/32-channel receive radiofrequency (RF) head coil (Nova Medical Inc., Wilmington, USA) was used. The experimental protocols were approved by the corresponding local ethics committees (“Commissie Mensgebonden Onderzoek regio Arnhem—Nijmegen” for measurements at 1.5 T and 3 T at the Donders Institute, Nijmegen, the Netherlands. The “Klinisches Ethik-Komitee” of the Universitätsklinikum Essen for measurements at 7 T at the Erwin L. Hahn Institute, Essen, Germany). Prior to scanning, all participants gave written informed consent in accordance with the guidelines of the local ethics committees.

### Data acquisition

The session protocol consisted of one high-resolution anatomical scan and one high-resolution functional scan, preceded by a low-resolution functional localizer. The purpose of the latter was to position the thin high-resolution functional slab to cover as much as possible of the primary visual cortex, V1, which would activate in response to the stimulus used.

Whole brain, high-resolution, T1-weighted anatomical scans were acquired using the MPRAGE sequence at 1.5 T and 3 T and the MP2RAGE at 7 T. Functional data for each subject were sampled with the cortical segmentation obtained from the corresponding anatomical scan at 3 T. The relevant parameters of the MPRAGE at 3 T were: TR/TI/TE = 2300/1100/3.15 ms, FA = 8°, voxel size = 0.8 mm isotropic, GRAPPA factor = 2. The anatomical scans at 1.5 T and 7 T were used to calculate the co-registration parameters between the functional scan and the anatomical scan at 3 T. The acquisition parameters for these sequences are given in SI:Table 1.

The functional online localizer consisted of acquiring a 2D-EPI sequence while the subject attended to the same stimulus pattern as in the subsequent high-resolution functional scan. Functional localizers were processed online and a map of voxels that responded to the stimulus was obtained. This map was used when positioning the thin slab in the high-resolution functional scan to guarantee that it maximally overlapped with the region responding to the stimulus. The relevant parameters of the functional localizer were the same across field strengths (e.g. 3.0 mm isotropic voxel size, TR = 3000 ms, FA = 90, 60 measurements), and the TEs were 44 ms, 30 ms, 30 ms at 1.5 T, 3 T and 7 T, respectively. The paradigm used during the functional localizer was 15 s of grey screen with a fixation cross, followed by 3 s of the same flickering checkerboard as in the functional scan.

The high-resolution functional scan was acquired using a multi-echo 3D-FLASH sequence with parameters (e.g. voxel size and echo spacing/range) matched across fields as far as possible (see Table 1 for sequence parameters). The slab thickness was thinner at 1.5 T and 3 T in order to minimise the acquisition time per volume or volume TR. Compared to 7 T, sequence TRs were longer at the two lower fields. These were limited by the last acquired echo, which was chosen based on literature values of T2* in grey matter (c.f. SI table 3a), and lengthened the acquisition. Besides, in-plane acceleration factors achievable with good image quality were lower than at 7 T (R = 3 instead of R = 4) for the lower fields^27,28^. In consequence, the slab thickness at these fields was reduced in order to reduce the acquisition time per volume and acquire enough volumes for a functional analysis on a reasonable time-scale.

**Table 1:** Relevant acquisition parameters at the three studied fields.

	1.5 T	3 T	7 T
**Functional scan**			
Sequence	3D-FLASH	3D-FLASH	3D-FLASH
Voxel size	0.75 mm isotropic	0.75 mm isotropic	0.75 mm isotropic
Slab thickness	12 mm	12 mm	16.5 mm
Nr of slices in the slab	16	16	22
BW/pixel	110 Hz	170 Hz	240 Hz
TE (10 echoes)	TE1/ΔTE/TE10 = 7.3/10.6/102.6 ms	TE1/ΔTE/TE10 = 5.9/8.1/79.0 ms	TE1/ΔTE/TE10 = 4.8/5.7/56.1 ms
Read-out gradient mode	Monopolar	Monopolar	Monopolar
TR	115 ms	95 ms	63 ms
Volume TR	160 s	130 s	97 s
Duration of functional paradigm	26 min 41 s	26 min 27 s	26 min 23 s
Number of volumes	10	12	18
FA	25°	20°	20°
Parallel Imaging (in plane)	3 (ref lines: 36)	3 (ref lines: 36)	4 (ref lines: 24)
**Anatomical scan**	MPRAGE with 1 mm isotropic voxel size	MPRAGE with 0.75 mm isotropic voxel size	MP2RAGE with 0.75 mm isotropic voxel size

The stimulation paradigm consisted of a circular checkerboard flickering at 7.5 Hz interleaved with a grey screen. Both conditions had a fixation cross in the middle. The duration of the stimulus block was equal to one volume acquisition time at each field (i.e. 160 s, 130 s and 97 s at 1.5 T, 3 T and 7 T respectively). The total acquisition time of the functional protocol was the same across B_0_ (~ 27 min), which allows the comparison of the fMRI performance in the same amount of time (but different numbers of stimulus cycles).

The BOLD response as a function of stimulus duration was studied during piloting to exclude any adaptation effect during the long stimulus ON block in the high-resolution functional acquisition. This was tested at 3 T using one subject and a 2D-EPI sequence, with 3.5 mm isotropic voxel size and TR/TE = 2000/30 ms. In this test, the following intra-stimulus patterns were studied: 6 s on–2 s off, 5 s on–3 s off, 7 s on–1 s off and 8 s continuously on. Each of the stimulus patterns was repeated 8 times, forming a stimulus ON block with a duration of 1 min 4 s. A stimulus ON block was followed by a grey screen with a fixation cross of 1 min 4 s and the sequence was repeated three times. This paradigm was run four times, once per intra-stimulus pattern tested, and the temporal mean and standard deviation of the BOLD response were examined for each of the stimulus-ON patterns. The continuously ON stimulus scored the highest mean BOLD signal with the lowest standard deviation, consequently this was the stimulus mode chosen for the study.

### Data processing

#### Motion correction and co-registration

First, the outer slices at the edges of the 3D functional slab were discarded due to imperfections of the slab profile. The skull was removed using FSL-BET^29^ and all echoes at a given time-point were summed to improve the SNR of the images prior to motion correction. Motion correction was performed in AFNI^30^ using a linearised weighted least squares algorithm (two passes, zero padded, 7th order polynomial interpolation), realigning all volumes to the first, and high-pass filtered with $f_{c} = 1/(2.1 \times volTR)$. Due to the very thin slab, some of the slices close to the edges suffered from lower or uneven signal intensity in the signal time course due to the interpolation when transforming the volume according to the motion correction parameters. Such slices were removed before further processing. The anatomical volume acquired at 3 T was co-registered to the first functional scan using the boundary based registration method implemented in FreeSurfer^31^.

#### Cortical sampling

The white matter surface was generated in FreeSurfer^10^ and it was expanded by 10% of the cortical depth at each vertex to sample the functional volume (15 sampling points across the cortex: 2 points in white matter, 11 points in the cortex and 2 points in the CSF)^32^. Segmentation performance was visually inspected and corrected in case of major failures. To avoid segmentation inaccuracies less obvious to the naked eye, vertices in V1 areas with an estimated cortical thickness larger than 2.5 mm were discarded from further analysis.

### Feature extraction

Five out of the twenty-seven scans acquired had to be discarded due to within-volume motion artefacts (two at 1.5 T and three at 7 T), and one scan due to deficient shimming in the occipital lobe. Features were extracted from the remaining twenty-one scans.

#### Signal extraction across the cortex

Depth resolved profiles were obtained by sampling unsmoothed functional volumes using nearest neighbour interpolation with the surface layering obtained in the anatomical scans (see subsection “Cortical sampling” above). All voxels sampled at a given depth were integrated over the corresponding region of interest and the average resting and activation signal over volumes were calculated. The region of interest consisted of:

- only activated voxels for metrics related to activation
- all V1 voxels that fell within the slab for metrics related to noise characteristics

#### Mask of activated voxels

**Figure 1:**
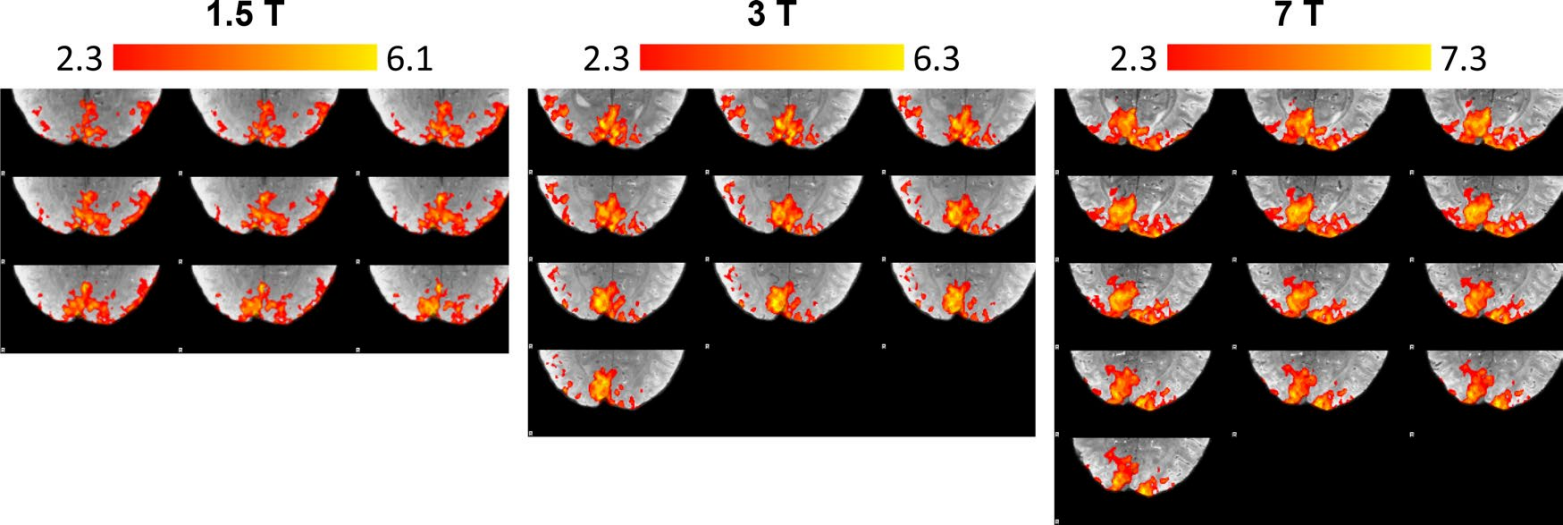
Exemplary activation maps obtained for the same subject at the three different static magnetic field strengths used. Significantly activated voxels are shown in colour. Those in primary visual cortex were used as a mask to extract activation profiles.

The selection of voxels responding to the stimulus was performed by using FSL-FEAT on the high-resolution data. After summing over all 10 echoes and motion correcting the data, a 3 mm smoothing kernel was applied and voxels that scored z > 2.3 were selected for the mask (see Fig. 1). Data were smoothed to ensure that the assumptions of random field theory, used to correct for multiple comparisons, were fulfilled, and to improve the sensitivity to activation in acquisitions with a poorer SNR. The surface boundaries obtained in FreeSurfer were overlaid onto the activation maps and vertices falling on activated voxels were identified. This activation map was used to extract the activation related signal for all echo times.

#### Feature estimation

R2* was calculated by fitting a mono-exponential function to the signal decay obtained for each cortical depth and volume. The average R2*-values at rest and activation were obtained by averaging over the corresponding state and ∆R2* was obtained as the difference of these two values.

BOLD signal measures were obtained by computing first the average signal in the region of interest per volume and cortical depth and calculating the BOLD signal change and the functional Contrast to Noise Ratio (CNR) per echo time thereafter (a summary of these well-known calculations is given in SI:Table 2).

#### Weisskoff test and physiological noise

The Weisskoff test was described in^33^ as a means of measuring scanner stability. This was performed by measuring, in a phantom, the temporal variance of the mean signal in ROIs of different sizes. The authors in^8^ applied this principle to estimate the minimum extent of the ROI that was needed until physiological noise became the dominant noise source. In order to measure this, an increasing number of voxels from 1 to N, where N is the maximum number of voxels in the area of interest, were averaged, and the temporal standard deviation of the average signal was computed. In the thermal noise dominated regime, this curve is expected to be proportional to 1/√N. If increasing the number of voxels added does not result in a decrease in the standard deviation over time, then the physiological noise dominated regime has been reached.

The Weisskoff-test was carried out by randomly selecting voxels within V1 that were added in each iteration until all voxels belonging to V1 within the slab were part of the ROI. The voxel intensity in an image is in arbitrary units. In order to be able to compare between fields, the standard deviation was normalized using the average signal of all voxels over the complete ROI for TE = T2*_GM_ at each field (Eq. 1):

1 $$\sigma_{{n_{i,TE} }} = \frac{{std_{i,TE} }}{{avg\left( {S_{{N,T2_{GM}^{*} }} } \right)}}$$

where *i* is the running index that voxels are averaged over before calculating the standard deviation over time, *TE* is the echo time, *S* is the magnitude of the voxel signal and *N* is the number of voxels in the primary visual cortex contained in the slab after motion correction.

## Results

### GE-BOLD signal change across the cortex

**Figure 2:**
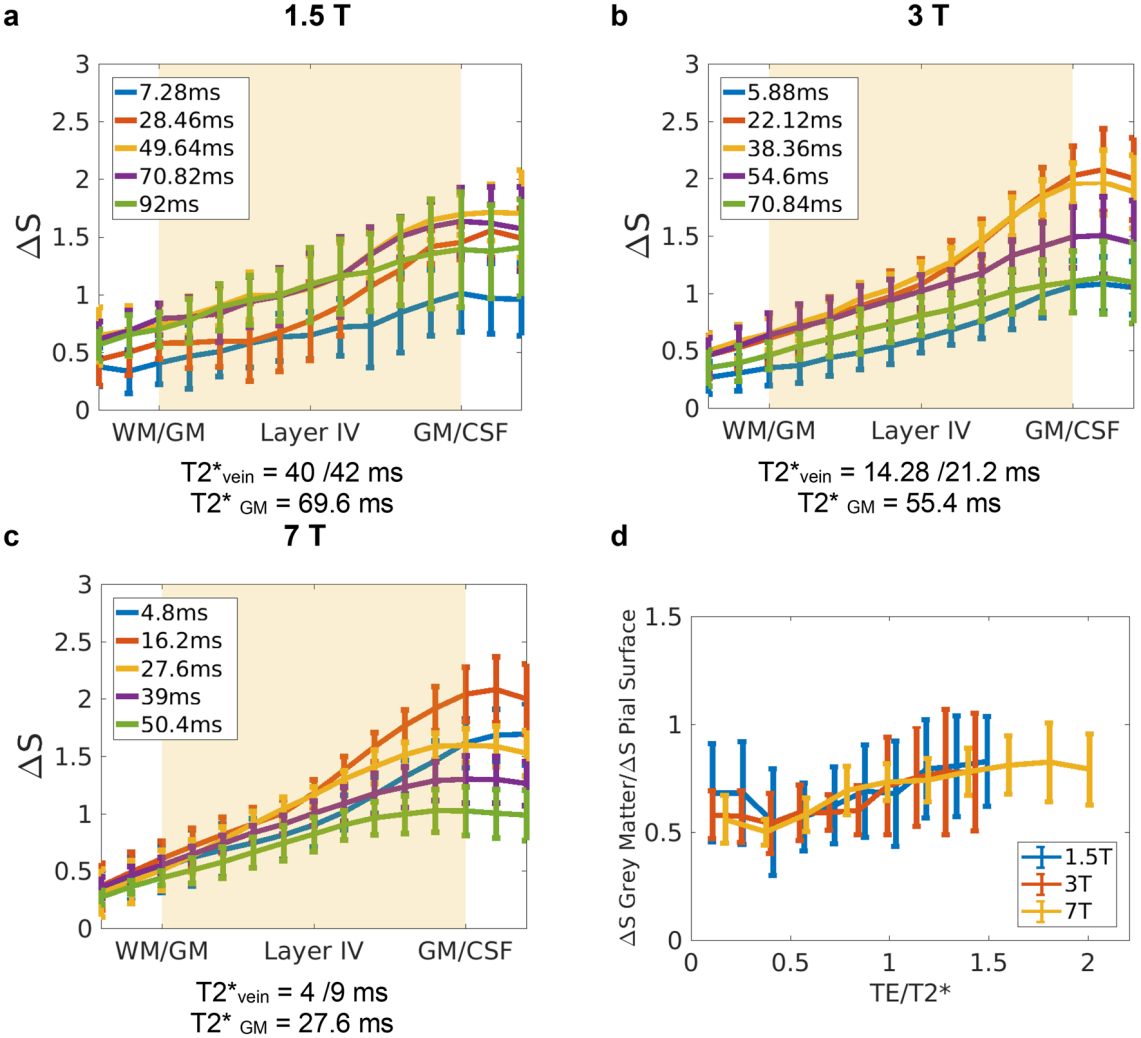
(**a**–**c**) BOLD signal change, ∆S, profiles averaged over subjects for different echo times (5 out of 10 echo times shown for visualization purposes). at 1.5 T, 3 T and 7 T (**a**), (**b**) and (**c**), respectively. ∆S is normalised to the mean signal change averaged over depth and TE per subject. The individual profiles have been divided by the mean value over echoes and cortex (hence, one value per profile), so that the average profile is not dominated by differences in the mean response between subjects. (**d**) The ratio between the signal change in the middle of the cortex and on the pial surface across fields. The x-axis shows echo times at each of the field strengths normalized by the corresponding T2* in the middle of the cortex. The 2-way ANOVA significance test shows that only the effect of the echo time was significant (p = 0.00). For short echo times (TE1–5), with considerable intravascular contribution, the grey matter to pial ratio is significantly different to that of late echoes (TE8–10), where venous signal has faded away.

∆S profiles in Fig. 2a–c show the characteristic ascending shape of GE-BOLD activation curves across the cortex^8,10,25,34–39^. This ascending pattern is believed to be the result of signal spread through intracortical veins from lower layers^40^ and partial volume effects with pial veins, which will make these profiles steeper close to the cortical surface.

The curves in Fig. 2a–c have been divided by the mean value of the signal change over echoes and cortex (i.e. one value per profile), so that the average profile is not dominated by differences in the mean response between subjects. This way, it is possible to focus on the differences in the activation profiles across echoes and fields. The profiles obtained at 1.5 T (Fig. 2a), which represent to our knowledge the first layer specific functional studies at this field, show qualitatively the same expected behaviour as those obtained at higher fields (Fig. 2b,c) but with a greater variance. Laminar GE-BOLD profiles at 3 T are less frequent in the literature. Nonetheless the results shown here (Fig. 2b) are in line with the ascending profiles for similar stimuli reported previously for this field strength^39,41^. The profiles at 7 T (Fig. 2c) are similar to those shown in^8^, only smoother. This is because the processing step used here did not involve manual realignment of the individual profiles to account for inaccurate registration, or segmentation of grey matter boundaries^8^.

Within a region, the steepness of the profiles depends on the intra- and extravascular signal contribution to the BOLD signal change, which varies with TE and B_0_. For example, at 7 T, the signal on the pial surface is larger for shorter TEs (compare yellow and orange curves). This is because at short echo times, there is still a venous intravascular contribution to the BOLD signal change (a compilation of venous relaxation times at different static field strengths is given in SI:Table 3b). This contribution is greater for large pial vessels on the pial surface than for venous vessels in the grey matter, due to the higher blood volume.

To summarise the data presented in Fig. 2a–c, and to allow a simpler comparison between fields, the grey matter to pial ratio of the cortical BOLD signal change was computed (Fig. 2d). This was calculated as the ratio between the signal change in the middle of the cortex (which corresponded to the 6^th^ cortical ’layer’ out of the 11 such layers defined in the cortex by FreeSurfer between white matter and CSF) to the signal change on the pial surface (11th layer in the cortex, which corresponds to the CSF/GM boundary). Note that in the x-axis in this graph TEs—which were different across fields-, have been normalised by the average T2* within the grey matter obtained at that field strength (c.f*.* SI table 3a). For equivalent echo times between fields, the magnitudes of these ratios are similar, around 0.65 for $TE = T_{{2_{GM} }}^{*}$. This similarity of the ratios is in line with the simulation results for the ratio of micro- and macrovascular contributions between fields given in^42^. A 2-way ANOVA test of the pial to grey matter ratio was performed to assess the effects of echo time, field strength, and their interaction. Only the effect of the echo time was significant. For short echo times (TE1-5), with considerable intravascular contribution, the grey matter to pial ratio is significantly different to that of late echoes (TE8-10), where venous signal has faded away.

### Resting R2* and ΔR2* upon activation

**Figure 3:**
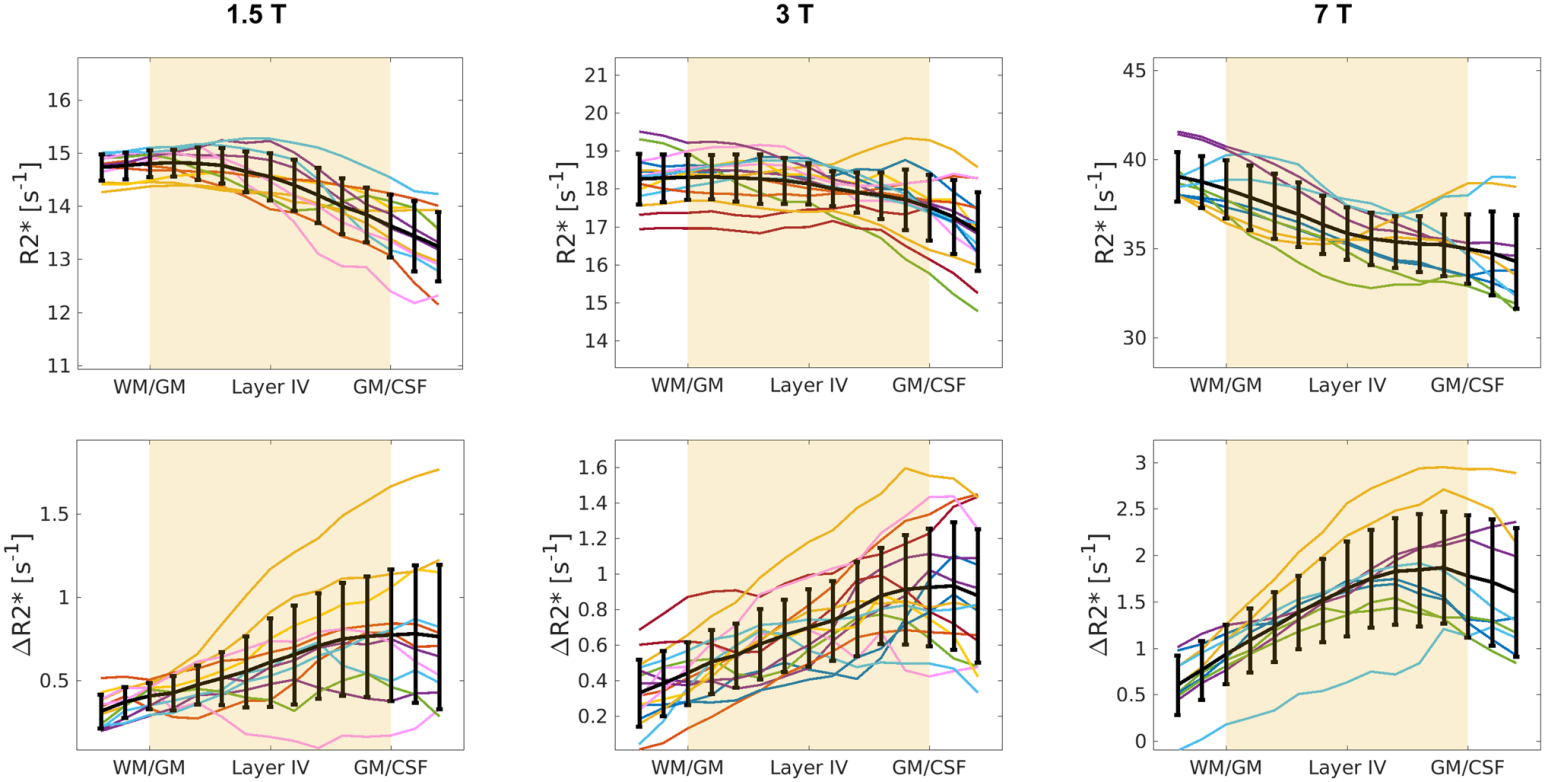
Individual R2* (at rest, upper row) and ∆R2* (during activation, bottom row) profiles for each subject and hemisphere (each colour corresponds to a subject and each line to the profile within a hemisphere). The black line is the mean ± std over subjects, normalized by the average T2* of grey matter at that field.

Transverse relaxation times at rest and upon activation are shown in Fig. 3. The acquisition and analysis approach used in this work (i.e. acquiring at submillimetre isotropic resolution and integrating over a given cortical depth) allows for a good separation between cortical signal and signal from the pial surface, as it limits the partial volume effects.

The mono-exponential signal decay was a good fit for the signal decay within grey matter. The estimated average T2* relaxation times within the cortex (T2* = 70, 55 and 27 ms approximately, see Table 2) are in line with those published previously (as summarised in SI:Table 3a). The average R2* values and profiles obtained at 7 T are similar to those shown in^43^. The reduction in R2* from the WM to the pial surface (Fig. 3a–c) was observed at all field strengths and has been attributed to a combination of reduced myelin and iron^44^. On the other hand, the variation in transverse relaxation rate upon activation, ΔR2*, showed the opposite behaviour, increasing from the WM to the pial surface, reflecting the increased blood partial volume and oxygenated blood draining towards the cortical surface.

**Table 2:** Comparison of basic activation related results across field strengths.

	1.5 T	3 T	7 T
Number of voxels in the slab after motion correction that fall in the middle of GM	1199 ± 401	1140 ± 342	1514 ± 278
% activated voxels in V1 within the slab	32%	34%	41%
TE of ∆S_max_ on the pial surface	39.05 ms	30.24 ms	10.5 ms
TE of ∆S_max_ in the middle of the cortex	60.23 ms	46.48 ms	21.9 ms
T2* in grey matter at rest	68.8 ± 2.1 ms	55.2 ± 1.7 ms	27.9 ± 1.2 ms

The voxels that coincide with the pial surface generated in FreeSurfer will in general contain three compartments with very different relaxation times: grey matter, CSF and pial veins. The mono-exponential fit to this signal would over-estimate the change of the relaxation time in the voxel. Unfortunately, it was not possible to fit a multi-compartment relaxation given the noise level in the data, the differences in the relaxation times of the compartments in the voxel, and the range and number of echo times used in this work.

### Temporal fluctuations of the resting signal

**Figure 4:**
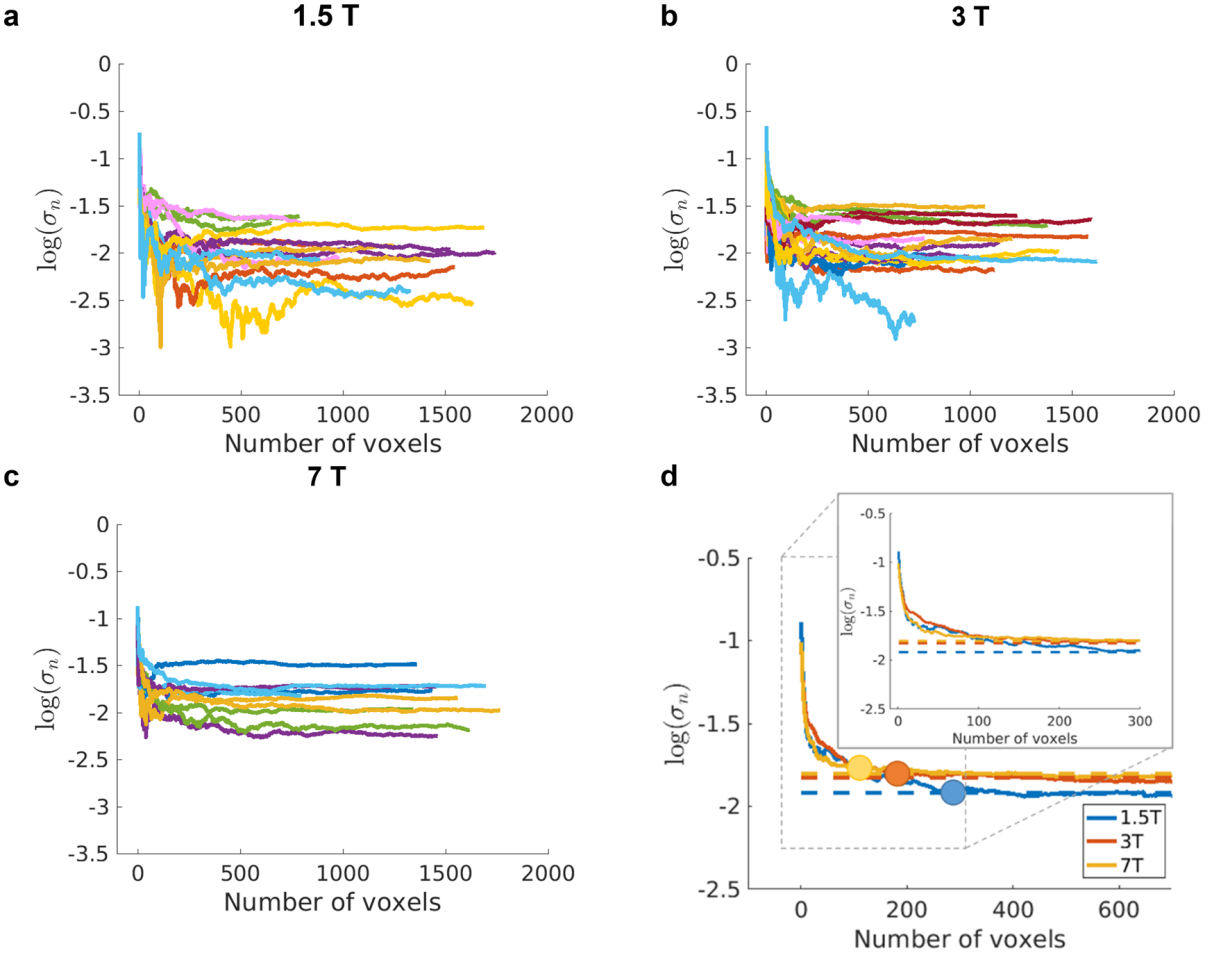
Results of the Weisskoff test: standard deviation over time of the mean resting signal as a function of the number of voxels averaged (see Eq. 1) at 1.5 T, 3 T and 7 T (**a**), (**b**) and (**c**), respectively. The voxels added were located in the middle of the cortex and the curves here correspond to TE = T2*_GM_ at each field. Each colour represents a subject, one line per hemisphere. The differences between the physiological noise levels are not significantly different (p = 0.49) between field strengths. (**d**) The average of the Weisskoff curves over subjects for the three fields studied. The circles correspond to the point in which the curve deviates from the plateau corresponding to the noise level where the physiological noise dominated regime has been reached (dashed lines with same colour-coding as for static field strength). The inset corresponds to the result for the first 300 voxels (without the circles).

Figure 4a–c shows the Weisskoff curves calculated as in Eq. (1) are shown in for all subjects and field strengths at TE ~ T2*_GM_. All Weisskoff curves reach the plateau related to the physiological noise dominated regime after integrating over maximally 250 voxels (about 100 mm^3^), except for one outlier at 1.5 T and 3 T. The differences in the signal to physiological noise ratio between fields (which corresponds to the plateau in the curves) are not statistically significant. A regression analysis between the magnitude of the physiological noise level and the motion parameters from the motion correction algorithm was performed and there was no relationship between the two: scatter plots showed a near random spread of data points (data not shown). Therefore, the variability in the physiological noise level can be related to the variability in the BOLD signal between sessions or/and to other sources of signal fluctuations that cannot be corrected in the post-processing, such as minimal movement during volume acquisition or small differences in the GRAPPA kernel used in each volume. These effects are expected to affect the noise level in the physiological noise dominated regime in a similar way at the three fields.

Figure 4d shows the average over subjects of the Weisskoff curves at each field strength, showing how progressive averaging ultimately leads to a plateau in the normalised standard deviation of the resting signal, σ_n_. Visual inspection shows that the point at which the average Weisskoff curve deviates from the noise level in the physiological noise dominated regime (dashed lines), is reached earlier with increasing field, but within 250 voxels at all fields [~ 100 voxels at 7 T (40 mm^3^), ~ 150 voxels at 3 T (60 mm^3^) and ~ 250 voxels at 1.5 T (100 mm^3^)].

**Figure 5:**
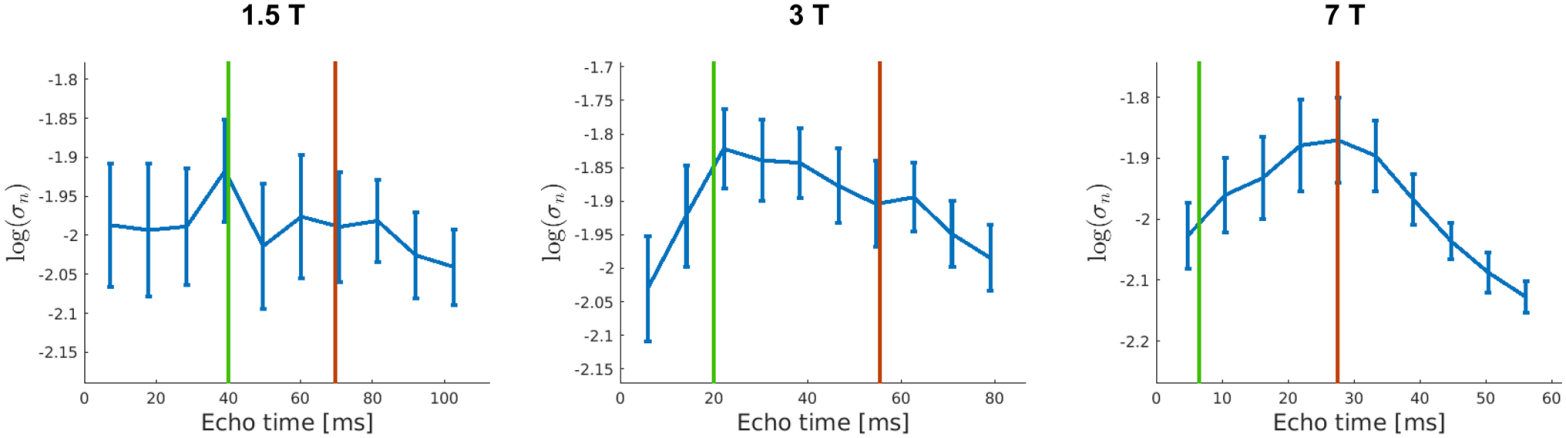
Average physiological noise level over subjects and standard error for the studied echo times in the middle of the cortex at 1.5 T (left), 3 T (middle) and 7 T (right). The green vertical line marks the venous T2* and the red vertical line marks the T2* of tissue at each field. Only the curve at 7 T is significantly different from a flat line (p = 0.009).

Figure 5 shows how the magnitude of the plateau of the Weisskoff curves in Fig. 4 varies as a function of TE. Following the physiological noise model in^16^, the non-BOLD like physiological noise (e.g. cardiac/respiratory fluctuations) scales with signal strength and will therefore decrease with echo time. The BOLD-like noise (e.g. resting neuronal fluctuations or blood flow changes), on the contrary, will follow the BOLD signal pattern. Figure 5 shows no consistent decrease in noise with TE indicating that BOLD-like physiological noise dominates. Although data have not been corrected for non-BOLD like noise due to the very long volume TRs, the results suggest that BOLD-like noise is the main contributor to physiological noise in this dataset. As a further check, the curves in Fig. 5 were compared to those obtained from single voxels, which varied randomly, as expected from a thermal noise dominated distribution.

The 1-way ANOVA test shows that the curves only display a statistically significant dependence of TE at 7 T. However, it is worth mentioning that the profiles for 1.5 T and 3 T show a higher noise level for echo times close to the T2* time of venous blood, marked in green in the figures, than for the echo time corresponding to T2*_GM_, marked in red. This pattern is reversed for the profile at 7 T, which shows a larger magnitude of the fluctuations at around the T2* of tissue. This is in line with the fact that intravascular contribution to the BOLD signal change is larger at 1.5 T and 3 T than at 7 T^45^.

### Functional contrast to noise ratio

**Figure 6:**
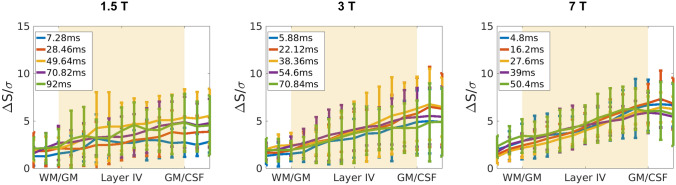
Functional CNR profiles at 1.5 T (left), 3 T (middle) and 7 T (right). The signal change upon activation (∆S), is divided by the standard deviation over time of the signal in the resting condition, σ. Profiles correspond to average ± std over subjects for different echo times (5 out of 10 echo times shown for visualization purposes). The CNR values in the middle of the parenchyma are not significantly different between 3 and 7 T (p-values ≥ 0.03, threshold corrected for false discovery error rate).

Figure 6 shows the functional contrast to noise ratio (CNR) across the cortex obtained after integrating the signal. The CNR is defined as the signal change upon activation (shown in Fig. 2a–c) divided by the temporal standard deviation over time at rest. This is considered a measure of the functional sensitivity. As in Fig. 2, the profiles are less smooth at 1.5 T, most likely because of poorer co-registration caused by greater difficulties in identifying the GM/WM boundary in the functional scan (refer to Fig. 1 for the reduced tissue contrast of the functional scan at 1.5 T). The magnitudes of the curves are similar, especially between 3 and 7 T, as can be expected when physiological noise is the dominant noise source in the signal (see “Discussion”). The error-bars at 3 T are larger than at 7 T but, if CNR profiles for the first 12 volumes at 7 T are calculated, the size of the error-bars increases (data not shown), indicating that the difference is mainly caused by the higher number of measurements performed per unit time at 7 T. A 2-way ANOVA test with interaction effects shows that within a field and in the middle of the cortex, there is no statistically significant TE-dependence of CNR. The differences in CNR are statistically significant between 1.5 T and 7 T. There is no interaction effect between field strength and echo time. Functional CNR for TE1 to TE3 at 1.5 T are significantly lower than for all TEs at 7 T (p-values = 0.0292 ± 0.0152).

**Figure 7:**
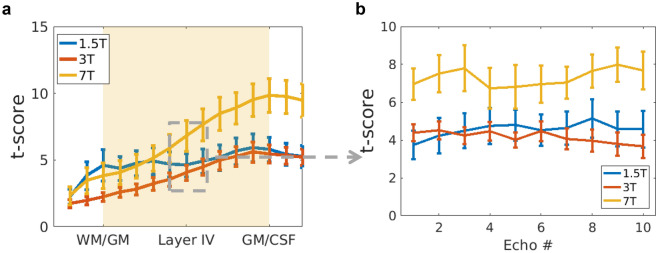
Average over subjects and standard error of (**a**) *t*-scores across the cortex for TE = T2*_GM_**,** and (**b**) t-scores in the middle of the cortex for each of the 10 echoes acquired. Note that the echo times acquired are different for the different static field strengths (see Table 1).

Despite the functional CNR being very similar between 3 and 7 T, Fig. 7a shows that the t-scores obtained at 7 T are higher at all echoes and cortical depths. The temporal resolution is higher at 7 T and more volumes are acquired per unit time, increasing the degrees of freedom of the statistic and therefore the t-score. Within a field strength, the *t*-scores obtained do not vary considerably with TE (Fig. 7b). In fact, a 2-way ANOVA test accounting for the interaction effect shows that there is no TE nor interaction effect of TE with field strength. The t-scores obtained at 7 T are significantly different to those obtained at 1.5 T and 3 T.

### Mask of activated voxels

Figure 1 shows the activation mask obtained at the three field strengths for a sample subject. The extent of the activation is not identical across field strengths, but they all extend over a representative region of the primary visual cortex. Slight differences in the experimental settings or acquisition (e.g. orientation of the head, placement of the functional slab or visual angle to the screen) are expected to average out after integrating within these masks. A quantitative assessment of the extent of the activation over field strengths and subjects is given in Table 2. The extent of the region responding to the stimulus in the primary visual cortex increases with field strength. As the masks were obtained using the same threshold for the z-score (2.3), the degrees of freedom when performing the statistical inference are higher at 7 T, the z-values will be increased, and more voxels will be above the defined threshold. In addition, the spatial coverage of V1 before and after motion correction was larger at 7 T.

If, instead of using activation maps to extract activation profiles, all of the primary visual cortex is considered, the pial to grey matter ratio delivers similar results (SI:Figure 1). The variation of ΔR2* in the grey matter across the field strengths follows the same trend when signal is integrated only over the region of V1 that shows activation and when it is integrated over all of V1. The magnitude of ΔR2* is smaller when all of V1 is considered, because cortex that is not responding to the activation is included too. This indicates that the masking step used covers an equally representative region at all field strengths and that it does not introduce a bias towards any of the fields.

## Discussion

### Scope of this work

This work studies the signal characteristics of layer specific GE-BOLD at 1.5 T, 3 T and 7 T, in combination with the commonly used post-processing technique of integrating, or otherwise combining, the depth-dependent signals within a region, to generate a single laminar profile. The GE-BOLD activation pattern depends both on the underlying vasculature, and the neuronal activity. The vascular response can vary over small patches of cortex, among other factors, due to the presence and size of the intracortical veins. As a result, the measured BOLD signal presents some spatial heterogeneity over small patches of cortex, as previously shown^46^. Our approach, and that of others, has generally been to identify activated regions on the basis of their aggregate response to the task, either by means of an independent activation study, or by smoothing the high resolution data and using this for a non-laminar analysis. Given that we are interested in the characteristics of the ROI as a whole^5,19,47^, an integration approach, or similar, represents the most logical strategy. Small variations caused by vasculature, or indeed gradients in functionality, will then not be accessible, and would require a different approach.

### Choice of static field strength

Our results indicate that the physiological noise limit will be reached for a volume of 40 mm^3^ at 7 T; 60 mm^3^ at 3 T; and 100 mm^3^ at 1.5 T. For the 0.75 mm voxels we used this corresponds to cortical areas of 53, 80, and 133 mm^2^ (lengths of 7.3, 8.9, and 11.5 mm) respectively. V1 has one of the thinnest cortices, and in other regions, with thicker laminae, the areas and lengths could be reduced while maintaining the same laminar resolution by increasing the voxel volume. In previous work in V1 we have used ROI volumes in the range 80–400 mm^3^^5,48,49^.

There may also be other considerations that also affect the choice of field strength. As mentioned in the Results the quality of co-registration was poorer at 1.5 T, which we believe reduced the smoothness of the laminar profiles. Furthermore, if voxels are selected for laminar analysis on the basis of single voxel statistics, for example orientation preference^48,49^ then discrimination will be superior at higher static field strengths because at the single voxel level thermal noise dominates, and poor voxel selection will negatively affect the quality of the laminar results. The lower acquisition efficiency at lower field strengths caused by the longer TE may also necessitate longer volume TRs or reduced volume coverage. These may be more important considerations for choosing the highest possible field strength than the sensitivity of the averaged laminar signal. The majority of laminar fMRI studies have to date been performed at 7 T, there have been a number of studies performed at 3 T and none at lower field strengths. With the increasing widespread availability of 7 T systems, we do not expect this picture to change in the future.

### Activation profiles and relaxation times

GE-BOLD activation profiles are similar for equivalent TEs at 3 T and 7 T. The profiles at 1.5 T show greater variance and a shallower incline going from white matter to CSF for short TEs. The incline is TE-dependent and this fact should be considered, as already mentioned in^8^, if profiles at different TEs or non-equivalent TEs are compared within or between fields.

The curves in Fig. 2d all have a minimum around 0.3–0.4 × T2*_GM_ which corresponds roughly to the T2* of venous blood. This shows that the use of shorter echo times increases the pial contribution of the measured signal change. At longer TEs the microvascular contribution to the signal steadily increases for the range of TEs considered in this study.

### Physiological noise dominated regime in the grey matter

The Weisskoff curves in Fig. 4 all reach a plateau, the state in which physiological noise is the dominant noise source. The average Weisskoff curves show that this regime is reached after averaging fewer voxels with increasing field strength; around 250, 150 and 100 voxels of 0.75^3^ mm^3^ at 1.5 T, 3 T and 7 T, respectively. An illustration of this for 250 voxels is given in SI:Figure 2.

Recently, as part of the Human connectome project, researchers were able to parcelate the cortex in 180 region per hemisphere^50^, resulting in an average surface per parcellated region of 333 mm^2^. Such a region is approximately twice as large as the patch found on this study for 1.5 T.

The reduction with increasing field strength in the volume of the cortex that it is required to integrate over to reach the physiological noise dominated regime is of course expected, as the physiological to thermal noise ratio increases with increasing static magnetic field strength^16^. Most of the improvement in the reduction of the temporal fluctuations is achieved after averaging a small number of voxels: for example, at 1.5 T, around 90% of the total reduction was achieved after averaging over 50 voxels (and sooner for the other two field strengths). These results are in line with the results shown in^14,15^ in which it is shown that physiological noise dominates even for voxels as small as 3 mm^3^ at 3 T and 7 T when a 32-channel array coil is used.

The integration approach along grey matter used in this work will lead to a higher tSNR as compared to the acquisition of a voxel with an equivalent volume (as long as the voxels have a significant grey matter fraction) as explained in^51^. An estimation of the increase in tSNR when the integration approach is used, is beyond the scope of this work, as it depends on the spatial extent of the correlation of the physiological noise over the studied region, which is not known.

The physiological noise profiles in Fig. 5 show that in this dataset BOLD-like noise is the dominant source of physiological noise. This dominance of BOLD-like noise is probably the result of considering pure grey matter voxels where the BOLD noise should be higher than in white matter or CSF^52^.

### Functional CNR when BOLD-like physiological noise dominates

The time-course CNR determines the functional sensitivity of a sequence and therefore sequence parameters are optimized to maximize this. The optimal TE depends on the dominant source of noise in the signal. If the MR signal in a voxel is accurately characterized by a mono-exponential decay plus some TE-independent noise, i.e., thermal noise, then the highest functional sensitivity or functional CNR will be achieved at TE = T2^(*)^. However, in the presence of physiological noise there will be a noise contribution that is TE dependent^16^. If the non-BOLD like physiological noise (i.e. mostly the corruption of the signal induced by the heart beat and respiration) dominates, then the maximum of the functional CNR curve will be located at TE > T2_GM_^(*)^^53^. If BOLD-like noise (i.e. signal fluctuations related to neural and hemodynamic fluctuations at rest) dominates, then the CNR curve will be flattened and the maximum functional sensitivity will be less TE-dependent^16,53^.

The CNR profiles across the cortex, see Fig. 6, are less steep than the ∆S profiles, especially for shorter TEs. This is because the shape and magnitude of the physiological noise profile as a function of TE follows to some extent that of ∆S, which results in a flattening of the profile and a convergence of the CNR value. In addition, the CNR-values, especially for 3 T and 7 T, are very similar. This implies that when physiological noise dominates, the signal properties of the functional response do not vary strongly with B0^16,54^. However, as the temporal resolution in the multi-echo protocols is higher at 7 T, the sequence is more efficient. This results in more degrees of freedom in the statistical analysis, leading to higher t-scores (Fig. 7a). As the CNR does not vary considerably between echo times, the t-scores obtained are also very similar between echoes for a given field strength (Fig. 7b).

### Laminar fMRI acquisition options when physiological noise dominates

It follows from Fig. 7b that, as long as BOLD-like physiological noise dominates, it would be possible to acquire at shorter TEs and improve the temporal resolution of the sequence. Furthermore, note that in the multi-echo acquisition presented in this work, the acquisition at longer TEs increases the TR.

The results in this study also suggest that, if the region of interest spreads over a larger area than the number of voxels required to be in the physiological noise dominated regime at 1.5 T (or 3 T), it should be possible to perform GE-BOLD laminar fMRI studies at field strengths lower than 7 T. Especially at 1.5 T, as the T2* contrast between white matter and grey matter is very poor at this field, it would be advantageous to acquire the functional data with a distortion matched T1-w anatomical scan, as this will considerably ease the segmentation and coregistration of the region^55–57^.

Lastly, for activated regions that are well within the physiological noise regime, it may be possible to increase the spatial resolution, which will potentially allow to differentiate more layers without sacrificing CNR.

### Choice of the acquisition protocol

The primary aim of this study was to characterize BOLD signal profiles across fields and echo times. Therefore, it was important to obtain largely distortion- and dropout-free images to be able to make a comparison based on signal properties alone. Otherwise, the results would have depended on the success of correction-methods in the post-processing. This is why the multi-echo FLASH sequence was used for the functional scan, instead of the EPI approach, more common in functional scans. In addition, the multi-echo implementation in the FLASH sequence allows a more accurate characterisation of the transversal signal decay than would be obtained with multi-echo EPI.

The design of the protocols used was made such that they would be maximally SNR efficient (acquiring during the total TR time), using the same number of echo times, and covering a comparable range of values for TE/T2* of tissue. This was considered the fairest comparison that could be designed across different field strengths.

The Lorentzian line-broadening induced by the relaxation during acquisition is Δf = 1/πT2*. In grey matter, this equals 4.6 Hz, 5.8 Hz and 11.4 Hz, at 1.5 T, 3 T and 7 T, respectively. As the 3D-FLASH sequence was used, the BW per pixels in the PE direction is infinite whereas the BW/pixel-values in the RO direction were 110, 170 and 240 Hz/pixel for 1.5 T, 3 T and 7 T, respectively. These values are well above the linewidth, hence they are not expected to have considerable differences in the degree of blurring across fields.

The visual cortex was chosen because it is a well-characterised primary cortical region, in which strong BOLD responses to neuronal activation can be registered. The drawback of studying V1 is that it is rather thin, ~ 2 mm on average. Therefore, in order to acquire depth resolved signal in V1, the acquisition of submillimetre voxel sizes in the 3 spatial directions was essential (0.75 mm isotropic voxel sizes were used in this study).

All these choices (i.e. distortion free, multi-echo and high-resolution acquisition) provided a high spatial accuracy that came at the cost of temporal resolution (acquisition time per volume over 1 min 30 s at all fields). As the acquisition time per volume varied with field (160 s, 130 s and 97 s at 1.5 T, 3 T and 7 T, respectively), the choice made was to keep the total acquisition time of the functional protocol equal across fields (~ 27 min) and assess the performance in the same amount of time. Because the volume acquisition time at 1.5 T and 3 T was considerably longer, the number of resting volumes at these fields is substantially lower. This results in noisier profiles of the temporal fluctuations of the resting signal, and lower *t-*values for the activation.

### Validity of the results shown here for BOLD fMRI that use a standard temporal resolution

In order to avoid the typical EPI distortion and signal losses that would hinder comparison between fields and echoes, high-resolution functional BOLD data were acquired using the FLASH sequence. As a consequence, the temporal resolution in this work is very poor compared to conventional fMRI studies. It is therefore worth discussing how the results obtained here apply to more common functional GE-BOLD acquisitions based on EPI.

The GE-BOLD profiles as a function of TE and static field strength are independent of the temporal resolution of the sequence^58^. reported that for stimuli longer than 20 s the venous compartment dilated, which was not the case for shorter stimuli. The stimulus durations at the different fields used here (i.e. 1 volume TR) are well above that boundary. Therefore, venous contribution across the cortex may be different than those obtained with shorter stimuli. However, this will be the case at the three studied fields (as the stimulus duration is well above 20 s in all cases), so the comparison between fields is still valid.

In terms of the noise behaviour of the resting signal, the results presented here apply to the case in which BOLD-like physiological noise is the dominant source of noise. Depending on the temporal resolution and acquisition scheme (2D/3D) used, the physiological noise level and distribution might be different to that obtained in this work. In addition, the laminar signal obtained in this study is likely to have additional sources of variance, which will appear as “physiological noise”, such as uncorrected small movements within the long volume acquisition times, or the fact that the GRAPPA kernel is calculated for each volume, which might introduce small variations in the time-course.

In order to have an idea of what the physiological noise level is in a more standard fMRI protocol, the Weisskoff test was performed on a functional dataset obtained using a 3D-EPI acquisition at 7 T (other relevant parameters: volume TR ~ 4 s, 0.9 mm isotropic voxels and TE = 22 ms), as shown in SI:Figure 3. The tSNR of the 3D-EPI for the integrated signal in the middle of the cortex was 68, whereas the average at 7 T in the FLASH sequence for the corresponding echo time was 78.9. The profile and physiological noise level obtained for this subject is comparable to those obtained using the 3D-FLASH (none of the datasets have been corrected for physiological noise). As the 3D-EPI was acquired at a single echo time, it is not possible to assess which is the dominant source of physiological noise in this dataset. However, if non-BOLD like physiological noise removal techniques are successfully applied in these datasets, then the dominant noise source would still be BOLD-like physiological noise, and the results regarding functional sensitivity would be unaffected.

Although the behaviour of 3D-EPI with acquisition times above the cardiac and respiratory signal periods might show a similar noise pattern to the data acquired in this study, 2D-EPI acquisitions are likely to show a very different noise pattern when compared to multi-shot acquisitions^59–61^. Hence, the extent over which it is necessary to integrate might be larger than the one shown in this manuscript.

In summary, this work shows that GE-BOLD signal changes across the cortex following activation are TE-dependent and have comparable activation profiles between fields for equivalent echo times. When BOLD-like physiological noise dominates, which was the case in this study, the Contrast to Noise Ratio (CNR) profiles are largely echo time and field strength independent. This implies that high-resolution laminar fMRI studies can be acquired at TE different to T2*_GM_ and/or at B_0_ < 7 T with comparable CNR. Nonetheless, spatial resolution and the efficiency of the acquisition increases with increasing static magnetic field. Hence, in practice higher field strengths are to be preferred if available.

## Supplementary Information

Supplementary Information.

## Data Availability

The data that support the findings of this study are available from the corresponding author upon reasonable request.

## References

[1] Felleman DJ, Van Essen DC (1991). Distributed hierarchical processing in the primate cerebral cortex. Cereb. Cortex.

[2] Triantafyllou C (2005). Comparison of physiological noise at 1.5 T, 3 T and 7 T and optimization of fMRI acquisition parameters. Neuroimage.

[3] De Martino F (2013). Cortical depth dependent functional responses in humans at 7T: Improved specificity with 3D GRASE. PLoS ONE.

[4] Muckli L (2015). Contextual feedback to superficial layers of V1. Curr. Biol..

[5] Kok P, Bains LJ, van Mourik T, Norris DG, de Lange FP (2016). Selective activation of the deep layers of the human primary visual cortex by top-down feedback. Curr. Biol..

[6] Fracasso A, Luijten PR, Dumoulin SO, Petridou N (2018). Laminar imaging of positive and negative BOLD in human visual cortex at 7T. Neuroimage.

[7] Kemper VG (2015). Sub-millimeter T2 weighted fMRI at 7 T: Comparison of 3D-GRASE and 2D SE-EPI. Front. Neurosci..

[8] Koopmans PJ, Barth M, Orzada S, Norris DG (2011). Multi-echo fMRI of the cortical laminae in humans at 7 T. Neuroimage.

[9] Maass A (2014). Laminar activity in the hippocampus and entorhinal cortex related to novelty and episodic encoding. Nat. Commun..

[10] Polimeni JR, Fischl B, Greve DN, Wald LL (2010). Laminar analysis of 7 T BOLD using an imposed spatial activation pattern in human V1. Neuroimage.

[11] Siero JC (2015). Cortical depth dependence of the BOLD initial dip and poststimulus undershoot in human visual cortex at 7 Tesla. Magn. Reson. Med..

[12] Siero JC (2013). BOLD specificity and dynamics evaluated in humans at 7 T: Comparing gradient-echo and spin-echo hemodynamic responses. PLoS ONE.

[13] Siero JC, Petridou N, Hoogduin H, Luijten PR, Ramsey NF (2011). Cortical depth-dependent temporal dynamics of the BOLD response in the human brain. J. Cereb. Blood Flow Metab..

[14] Triantafyllou C, Polimeni JR, Keil B, Wald LL (2016). Coil-to-coil physiological noise correlations and their impact on functional MRI time-series signal-to-noise ratio. Magn. Reson. Med..

[15] Triantafyllou C, Polimeni JR, Wald LL (2011). Physiological noise and signal-to-noise ratio in fMRI with multi-channel array coils. Neuroimage.

[16] Krüger G, Glover GH (2001). Physiological noise in oxygenation-sensitive magnetic resonance imaging. Magn. Reson. Med..

[17] Hyde JS, Biswal BB, Jesmanowicz A (2001). High-resolution fMRI using multislice partial k-space GR-EPI with cubic voxels. Magn. Reson. Med..

[18] Guidi M, Huber L, Lampe L, Gauthier CJ, Moller HE (2016). Lamina-dependent calibrated BOLD response in human primary motor cortex. Neuroimage.

[19] Scheeringa R, Koopmans PJ, van Mourik T, Jensen O, Norris DG (2016). The relationship between oscillatory EEG activity and the laminar-specific BOLD signal. Proc. Natl. Acad. Sci. U.S.A..

[20] Kashyap S, Ivanov D, Havlicek M, Poser BA, Uludag K (2018). Impact of acquisition and analysis strategies on cortical depth-dependent fMRI. Neuroimage.

[21] Puckett AM, Aquino KM, Robinson PA, Breakspear M, Schira MM (2016). The spatiotemporal hemodynamic response function for depth-dependent functional imaging of human cortex. Neuroimage.

[22] Olman CA (2012). Layer-specific fMRI reflects different neuronal computations at different depths in human V1. PLoS ONE.

[23] Finn ES, Huber L, Jangraw DC, Molfese PJ, Bandettini PA (2019). Layer-dependent activity in human prefrontal cortex during working memory. Nat. Neurosci..

[24] Huber L (2017). High-resolution CBV-fMRI allows mapping of laminar activity and connectivity of cortical input and output in human M1. Neuron.

[25] Huber L (2015). Cortical lamina-dependent blood volume changes in human brain at 7 T. Neuroimage.

[26] Goa PE, Koopmans PJ, Poser BA, Barth M, Norris DG (2014). BOLD fMRI signal characteristics of S1- and S2-SSFP at 7 Tesla. Front. Neurosci..

[27] Ohliger MA, Grant AK, Sodickson DK (2003). Ultimate intrinsic signal-to-noise ratio for parallel MRI: Electromagnetic field considerations. Magn. Reson. Med..

[28] Wiesinger F, Boesiger P, Pruessmann KP (2004). Electrodynamics and ultimate SNR in parallel MR imaging. Magn. Reson. Med..

[29] Smith SM (2002). Fast robust automated brain extraction. Hum. Brain Mapp..

[30] Cox RW (1996). AFNI: Software for analysis and visualization of functional magnetic resonance neuroimages. Comput. Biomed. Res..

[31] Greve DN, Fischl B (2010). Accurate and robust brain image alignment using boundary-based registration. Neuroimage.

[32] Dale AM, Fischl B, Sereno MI (1999). Cortical surface-based analysis I. Segmentation and surface reconstruction. Neuroimage.

[33] Weisskoff RM (1996). Simple measurement of scanner stability for functional NMR imaging of activation in the brain. Magn. Reson. Med..

[34] Trampel R (2012). Laminar-specific fingerprints of different sensorimotor areas obtained during imagined and actual finger tapping. Proc. Int. Soc. Magn. Reson. Med..

[35] Duong TQ, Silva AC, Lee SP, Kim SG (2000). Functional MRI of calcium-dependent synaptic activity: Cross correlation with CBF and BOLD measurements. Magn. Reson. Med..

[36] Lu HB (2004). Spatial correlations of laminar BOLD and CBV responses to rat whisker stimulation with neuronal activity localized by Fos expression. Magn. Reson. Med..

[37] Goense JBM, Logothetis NK (2006). Laminar specificity in monkey V1 using high-resolution SE-fMRI. Magn. Reson. Imaging.

[38] Jin T, Kim SG (2008). Cortical layer-dependent dynamic blood oxygenation, cerebral blood flow and cerebral blood volume responses during visual stimulation. Neuroimage.

[39] Koopmans PJ, Barth M, Norris DG (2010). Layer-Specific BOLD activation in human VI. Hum. Brain Mapp..

[40] Markuerkiaga I, Barth M, Norris DG (2016). A cortical vascular model for examining the specificity of the laminar BOLD signal. Neuroimage.

[41] Ress D, Glover GH, Liu J, Wandell B (2007). Laminar profiles of functional activity in the human brain. Neuroimage.

[42] Uludag K, Muller-Bierl B, Ugurbil K (2009). An integrative model for neuronal activity-induced signal changes for gradient and spin echo functional imaging. Neuroimage.

[43] Marques JP, Khabipova D, Gruetter R (2017). Studying cyto and myeloarchitecture of the human cortex at ultra-high field with quantitative imaging: R1, R2* and magnetic susceptibility. Neuroimage.

[44] Stuber C (2014). Myelin and iron concentration in the human brain: A quantitative study of MRI contrast. Neuroimage.

[45] Cheng Y, van Zijl PCM, Hua J (2015). Measurement of parenchymal extravascular R2* and tissue oxygen extraction fraction using multi-echo vascular space occupancy MRI at 7 T. NMR Biomed..

[46] Kashyap S (2018). Resolving laminar activation in human V1 using ultra-high spatial resolution fMRI at 7T. Sci. Rep..

[47] Sharoh D (2019). Laminar specific fMRI reveals directed interactions in distributed networks during language processing. Proc. Natl. Acad. Sci. U.S.A..

[48] Lawrence, S. J. D., Norris, D. G. & de Lange, F. P. Diassociable laminar profiles of concurrent bottom-up and top-down modulation in the human visual cortex. *Elife* **8** (2019). 10.7554/eLife.44422 PMC6538372 31063127

[49] Lawrence SJD (2018). Laminar organization of working memory signals in human visual cortex. Curr. Biol..

[50] Glasser MF (2016). A multi-modal parcellation of human cerebral cortex. Nature.

[51] Triantafyllou C, Hoge RD, Wald LL (2006). Effect of spatial smoothing on physiological noise in high-resolution fMRI. Neuroimage.

[52] Bodurka J, Ye F, Petridou N, Murphy K, Bandettini PA (2007). Mapping the MRI voxel volume in which thermal noise matches physiological noise—Implications for fMRI. Neuroimage.

[53] Wald LL, Triantafyllou C, Hoge RD, Uludag K, Ugurbil K, Berliner L (2015). fMRI: From nuclear spins to brain functions.

[54] Ugurbil K, Robitaille P-M, Berliner L (2006). Ultra High Field Magnetic Resonance Imaging.

[55] Kashyap, S., Ivanov, D., Havlicek, M., Poser, B.A. & Uludag, K. in *Proceedings of the 24th Annual Meeting of the ISMRM*, Singapore (2016).

[56] van der Zwaag W (2018). Distortion-matched T1 maps and unbiased T1-weighted images as anatomical reference for high-resolution fMRI. Neuroimage.

[57] Huber, L. *et al*. in *Proceedings of the 24th Annual Meeting of the ISMRM*, Singapore (2016).

[58] Kim T, Kim S-G (2011). Temporal dynamics and spatial specificity of arterial and venous blood volume changes during visual stimulation: Implication for BOLD quantification. J. Cereb. Blood Flow Metab..

[59] Reynaud O, Jorge J, Gruetter R, Marques JP, van der Zwaag W (2017). Influence of physiological noise on accelerated 2D and 3D resting state functional MRI data at 7 T. Magn. Reson. Med..

[60] Huber L (2018). Techniques for blood volume fMRI with VASO: From low-resolution mapping towards sub-millimeter layer-dependent applications. Neuroimage.

[61] Lutti A, Thomas DL, Hutton C, Weiskopf N (2013). High-resolution functional MRI at 3 T: 3D/2D echo-planar imaging with optimized physiological noise correction. Magn. Reson. Med..

