# Supplementary material for: An in-vivo study of BOLD laminar responses as a function of echo time and static magnetic field strength
Source: Sci Rep. 2021 Jan 21;11:1862. doi: 10.1038/s41598-021-81249-w (PMC7820587; doi:10.1038/s41598-021-81249-w)
Supplement: Supplementary file 1 — Supplementary Information. [file 41598_2021_81249_MOESM1_ESM.pdf]

SUPPLEMENTARY INFORMATION FOR

An in-vivo study of BOLD laminar responses as a function of echo time and static magnetic field strength

Irati Markuerkiaga[1], José P. Marques[1], Lauren J. Bains[1], David G. Norris[1,2]

[1] Donders Centre for Cognitive Neuroimaging, Radboud University, Nijmegen, The Netherlands
[2.] Erwin L. Hahn Institute for Magnetic Resonance Imaging, 45141 Essen, Germany

SI:FIGURES

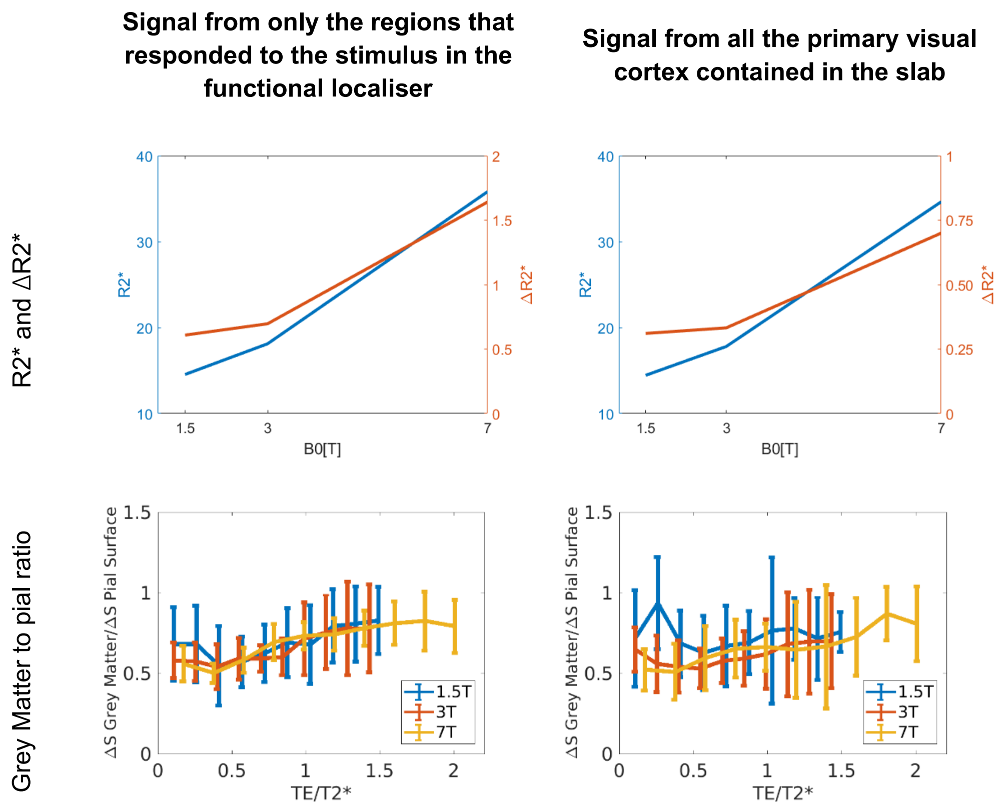

SI:Figure 1: Relaxation time values and changes, upper row, and parenchyma to pial ratios, lower row, obtained considering the signal only from the activation mask, left, like in the body of the manuscript and considering signal from all of the primary visual cortex contained in the slab, right.

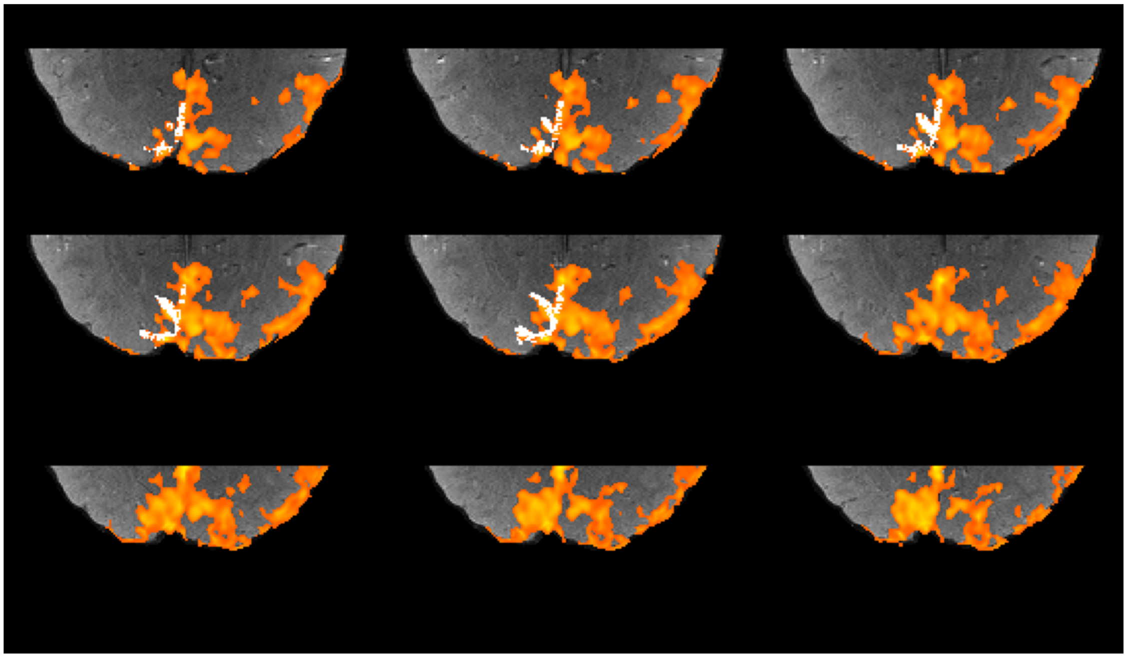

SI:Figure 2: The area in the occipital region that responded to the stimulus at 1.5T is shown in hot-body scale superimposed on the FLASH image (these are the same data as shown in the left column of Figure 1 in the manuscript). The white overlay shows 250 contiguous voxels chosen arbitrarily from the middle layer of the cortex. For visualisation purposes, also voxels within the grey matter located above and below the mid-cortical voxels are highlighted.

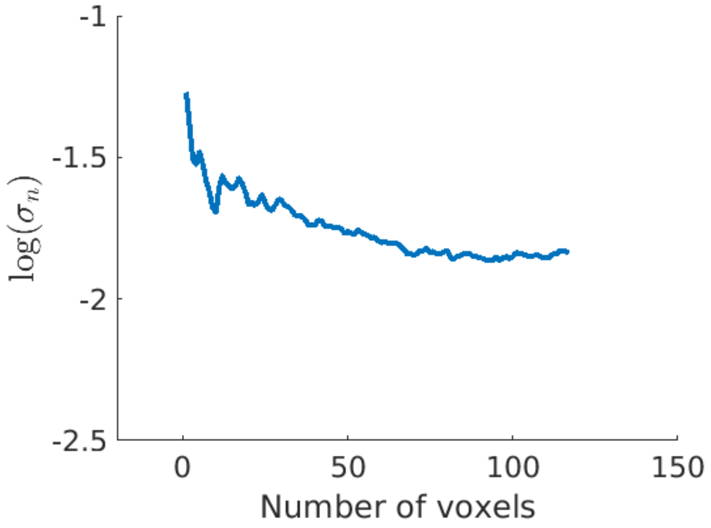

SI:Figure 3: Weisskoff test for 3D-EPI with 0.9mm isotropic voxel size and volume TR=3.96 s.

SI:TABLES

	**MPRAGE @ 1.5 T**	**MPRAGE @ 3 T**	**MP2RAGE @ 7 T**
TR	2300 ms	2300 ms	6000 ms
TI1/TI2	1100/-ms	1100/- ms	800 /2700 ms
TE	2.92 ms	3.15 ms	3.06 ms
FA1/FA2	8°/-	8°/-	4/5°
Voxel size	1x1x1 mm^3	0.8x0.8x0.8 mm^3	0.75x0.75x0.84 mm^3
GRAPPA acceleration factor	3	2	3
Acquisition time	4 min 31 s	6 min 35 s	9 min 38 s

SI:Table 1: Relevant parameters of the whole brain anatomical scans at each of the field strengths.

**Name**	**Abbreviation**	**Formula**
BOLD signal change	ΔS	$\Delta S = \overline{S_{act}} - \overline{S_{rest}}$
Functional contrast to noise ratio	(Functional) CNR	$CNR = (\overline{S_{act}} - \overline{S_{rest}})/std(S_{rest})$
t-score	t	$S = X\beta + \varepsilon$ $t = \frac{c'\beta}{\sqrt{Var(\varepsilon)c'(XX')^{-1}c}}$

SI:Table 2: List of activation related metrics and their functions used in this manuscript.

Area and paper	Voxel size [mm^3]	1.5 T	3 T	4T	7 T
**Occipital lobe** [1]	1x1x3	74.9	59.7		42.1
**Primary visual cortex**[2]	1.25x1.25x5	69.4		31.7	
**Visual cortex** [3]	0.75x0.75x5			41.4+/-5.5	25.1+/-3.5
**Average in cortical GM** [4*]	2.1x2.1x3	84.0+/-0.8	66.0+/-1.4		33.2+/-1.3

SI:Table 3a: An overview of $T2^*_{GM}$ values (in ms) reported in the literature.

Paper	1.5 T	3 T	7 T
**Y=0.6, Hct=0.43**[5]	40	14.28	4
**Y = 0.62, Hct=0.44**[6]		21.2	
**Y=0.72** [7]	42+/-2.8		
**Y = 0.45**[3]			9

SI:Table 3b: Venous T2* times, in ms) reported in the literature (ex-vivo samples). *Y* refers to the oxygenation of blood and *Hct* to hematocrit.

Bibliography (Supplementary Information)

1. Cox, E. & Gowland, P. Measuring T2 and T2' in the brain at 1.5T, 3T and 7T using a hybrid gradient echo-spin echo sequence and EPI. *Proc. 16th Sci. Meet. Int. Soc. Magn. Reson. Med.* **Toronto**, 1411 (2008).

2. Gati, J. S., Menon, R. S., Ugurbil, K. & Rutt, B. K. Experimental determination of the BOLD field strength dependence in vessels and tissue. *Magn. Reson. Med.* **38**, 296–302 (1997).

3. Yacoub, E. *et al.* Imaging Brain Function in Humans at 7 Tesla. **594**, 588–594 (2001).

4. Peters, A. M. *et al.* T2* measurements in human brain at 1.5, 3 and 7 T. *Magn. Reson. Imaging* **25**, 748–753 (2007).

5. Blockley, N. P. *et al.* Field strength dependence of R1 and R2* relaxivities of human whole blood to ProHance, Vasovist, and deoxyhemoglobin. *Magn. Reson. Med.* **60**, 1313–20 (2008).

6. Zhao, J. M., Clingman, C. S., Närväinen, M. J., Kauppinen, R. a & van Zijl, P. C. M. Oxygenation and hematocrit dependence of transverse relaxation rates of blood at 3T. *Magn. Reson. Med.* **58**, 592–7 (2007).

7. Barth M, Moser E. Proton NMR relaxation times of human blood samples at 1.5 T and implications for functional MRI. *Cell. Mol. Biol.* **43**, 783–791 (1997).
